# Evaluation of the FUSION-X-US-II prototype to combine automated breast ultrasound and tomosynthesis

**DOI:** 10.1007/s00330-020-07573-3

**Published:** 2020-12-12

**Authors:** Benedikt Schäfgen, Marija Juskic, Marcus Radicke, Madeleine Hertel, Richard Barr, André Pfob, Riku Togawa, Juliane Nees, Alexandra von Au, Sarah Fastner, Aba Harcos, Christina Gomez, Anne Stieber, Fabian Riedel, André Hennigs, Christof Sohn, Joerg Heil, Michael Golatta

**Affiliations:** 1 Department of Gynecology and Obstetrics, University Breast Unit, Heidelberg, Germany; 2 Siemens Healthcare GmbH, Forchheim, Germany; 3 Northeastern Ohio Medical University and Southwoods Imaging, Youngstown, OH USA; 4 Department of Radiology, University Breast Unit, Heidelberg, Germany

**Keywords:** Early detection of cancer, Ultrasonography, mammary, Mammography, Imaging, three-dimensional, Multimodal imaging

## Abstract

**Objective:**

The FUSION-X-US-II prototype was developed to combine 3D automated breast ultrasound (ABUS) and digital breast tomosynthesis in a single device. We evaluated the performance of ABUS and tomosynthesis in a single examination in a clinical setting.

**Methods:**

In this prospective feasibility study, digital breast tomosynthesis and ABUS were performed using the FUSION-X-US-II prototype without any change of the breast position in patients referred for clarification of breast lesions with an indication for tomosynthesis. The tomosynthesis and ABUS images of the prototype were interpreted independently from the clinical standard by a breast diagnostics specialist. Any detected lesion was classified using BI-RADS® scores, and results of the standard clinical routine workup (gold standard) were compared to the result of the separate evaluation of the prototype images. Image quality was rated subjectively and coverage of the breast was measured.

**Results:**

One hundred one patients received both ABUS and tomosynthesis using the prototype. The duration of the additional ABUS acquisition was 40 to 60 s. Breast coverage by ABUS was approximately 80.0%. ABUS image quality was rated as diagnostically useful in 86 of 101 cases (85.1%). Thirty-three of 34 malignant breast lesions (97.1%) were identified using the prototype.

**Conclusion:**

The FUSION-X-US-II prototype allows a fast ABUS scan in combination with digital breast tomosynthesis in a single device integrated in the clinical workflow. Malignant breast lesions can be localized accurately with direct correlation of ABUS and tomosynthesis images. The FUSION system shows the potential to improve breast cancer screening in the future after further technical improvements.

**Key Points:**

*• The FUSION-X-US-II prototype allows the combination of automated breast ultrasound and digital breast tomosynthesis in a single device without decompression of the breast.*

*• Image quality and coverage of ABUS are sufficient to accurately detect malignant breast lesions.*

*• If tomosynthesis and ABUS should become part of breast cancer screening, the combination of both techniques in one device could offer practical and logistic advantages. To evaluate a potential benefit of a combination of ABUS and tomosynthesis in screening-like settings, further studies are needed.*

## Introduction

Mammography is the basis of breast cancer screening worldwide. Early detection and treatment of breast cancer can result in reduced mortality [1–3]. Still there are limitations on mammography in certain patient groups. Dense breast tissue leads to a reduced sensitivity of mammography and is also an independent risk factor for developing breast cancer [4, 5]. In these patients, breast ultrasound as a supplement to mammography has been shown to detect 1.8 to 3.7/1000 additional malignancies [6–8]. However, traditional hand-held ultrasound (HHUS) is highly examiner-dependent and the examination and interpretation are time-consuming. 3D automated breast ultrasound (ABUS) has the potential to overcome these limitations [9–13].

Tomosynthesis, a 3D mammogram created from low-dose digital X-ray projections, has been shown to increase sensitivity and specificity of breast cancer detection compared to 2D-mammography [14–17]. Several studies have shown that ABUS and tomosynthesis can improve breast cancer screening in women with dense breast tissue [18–21]. There are few reports on hybrid devices combining ultrasound and mammography/tomosynthesis in a single device, including our evaluation of the FUSION-X-US-I prototype [22–27]. The first prototype yielded promising results but key issues were limited breast coverage of ABUS and insufficient evidence whether malignant lesions could be reliably detected [26]. The successor FUSION-X-US-II has now been developed with technical improvements. In the present study, we evaluate the detection and classification of breast lesions using the FUSION-X-US-II prototype in a clinical setting with patients at our breast unit.

## Material and methods

### Study design

This was a prospective single-center study at our breast unit performed in 2019. Patients were eligible for the study if they presented for clarification of breast lesions with an indication for tomosynthesis. Exclusion criteria were previous breast surgeries in the examined breast, pregnancy, age of less than 18 years, and inability to give informed consent. Patients were recruited non-selectively if the prototype and the instructed personnel were available. The standard diagnostic workup included clinical examination, 2D-digital mammography (MAMMOMAT Inspiration, Siemens Healthcare GmbH), HHUS using an ACUSON S2000 or S3000 ultrasound unit with an 18-MHz transducer (Siemens Healthcare GmbH), and tomosynthesis using the FUSION-X-US-II prototype (Siemens Healthcare GmbH). The study-specific additional ABUS was obtained in the same setting directly after tomosynthesis by a radiologic technologist. No sonographer or physician is needed to perform the examination [28]. An ultrasound-guided biopsy for histopathological confirmation was performed in 47 cases with BI-RADS® 4 or 5 according to the international guidelines [24]. The result of the whole standard diagnostic workup was defined as gold standard.

The study protocol was accepted by the appropriate ethics committee (Medical Faculty Heidelberg, reference number S-438/2018) in concordance with the Health Information Portability and Accountability Act of 1996 (HIPPA). Informed consent was obtained from every patient enrolled in the study.

### Equipment/imaging protocol

The FUSION-X-US-II prototype is based on the ACUSON S2000 Automated Breast Volume Scanner (Siemens Healthcare GmbH) and the MAMMOMAT Inspiration (Siemens Healthcare GmbH), which are both FDA approved and CE certified. The research device combining both is not commercially available. The prototype was developed from the FUSION-X-US-I prototype with several technical improvements. In comparison to the previous FUSION-X-US-I prototype, the compression paddle was adapted in terms of weight and size to provide better positioning. The breast is compressed using a specially woven gauze, which provides sufficient ultrasound coupling through more constant tension and equal pressure distribution for ABUS and X-ray permeability for tomosynthesis. An improved tightening mechanism of the gauze enables a more conform compression. A new feature to improve contact between the breast surface and the ultrasound probe has been implemented: A special air cushion attached to the housing of the X-ray detector can be inflated to push the peripheral parts of the breast homogenously towards the gauze. The inflation is controlled manually by the radiologic technologist.

Apart from the inflation of the air cushion, the performance of tomosynthesis including the positioning of the patient and compression of the breast does not differ from routine examinations. After tomosynthesis, the breast remains compressed in the same position until the ABUS scan is completed. ABUS and tomosynthesis images are transferred to a digital workstation where the corresponding coordinate systems are aligned so that both modalities are linked and can be analyzed side by side.

ABUS acquisition by the prototype differs from a regular ABUS by covering an increased area of maximum 30 × 15.4 cm^2^ with a maximum penetration depth of 10 cm, resulting in 585 slices for one volume. In comparison, standard ABUS covers an area of 16.8 × 15.4 cm^2^ with a penetration depth of 6 cm and 318 slices.

### Image analysis

In the study setting, a physician with more than 10 years of experience with ABUS systems evaluated the tomosynthesis and ABUS scans using the syngo.breast ultrasound software (Software Version VA25, ©2012-2013 Siemens Medical Solutions, Inc.) together with an additional software tool for side-by-side matching of ABUS and tomosynthesis slides (XUS Viewing Prototype, Siemens Healthcare GmbH). He was blinded to the results of the standard diagnostic workup. Image quality of ABUS was rated subjectively by the physician on a scale ranging from 1 to 5. Categories 1 and 2 represent a quality below diagnostic applicability without (category 1) or with (category 2) identifiable breast structures. Categories 3 to 5 depict a sufficient quality for diagnostic applicability with a quality that is lower than that in HHUS (category 3), close to/comparable to HHUS (category 4), and equal or higher compared to HHUS (category 5). All detected lesions were measured and classified using BI-RADS® scores (18). To quantify the breast coverage in ABUS and tomosynthesis, the depicted breast area at the level of the nipple region was measured using the software Fiji (ImageJ, Version 2.0.0, ©2010–2020) in both techniques. The breast coverage of tomosynthesis was used as a reference standard for the coverage of ABUS.

The primary endpoint was the breast cancer detection rate in the study setting. Secondary endpoints were the detection and classification of benign findings, image quality, breast coverage, and the time of performance and interpretation of ABUS and tomosynthesis.

### Statistical analysis

This explorative study is based on descriptive statistical methods. The *p* values are not corrected for multiplicity and must be interpreted descriptively. RStudio was used for statistical analysis (RStudio, Version 1.2.1335, Inc., © 2009–2019). Mean with standard deviation or median with quartiles are given for values with normal or non-normal data distribution. To assess the primary endpoint, we correlated the malignant breast lesions described in the standard diagnostic workup with the findings from the study setting and compared localization, size, and BI-RADS® classifications. The difference in quality over the projections was tested for significance with the two-sided Fisher’s exact test, and the difference in the breast coverage of tomosynthesis and ABUS was tested for significance with the Mann-Whitney *U* test. Significance was set as α = 0.05.

## Results

### Study cohort and technical function

One hundred fifty-two included patients received tomosynthesis using the prototype (for cohort description, see Table 1). In 51 of 152 cases (33.6%), the ABUS scan could not be completed due to software errors and hardware problems. The most frequent software errors were a failure to start the scan, early abort of the scan, and failure to transmit or save the ABUS data. Hardware problems, which could be identified and partly corrected in the course of the study, included defective electrical contacts at the interface of the ABUS system and the prototype hardware. If the scan could not be initiated right away, ABUS was not performed to avoid waiting time or prolonged compression for the patient. No scan had to be cancelled by the examiner for reasons of patient safety or tolerability.

**Table 1:** Cohort description

Number of patients*	101
Age (years)**	57.61 ± 11.98
BMI (kg/m^2^)**	26.72 ± 5.84
Cup size*	
A	7 (6.9)
B	30 (29.7)
C	12 (11.9)
D	17 (16.8)
E	6 (5.9)
F	2 (2.0)
n.a.	27 (26.7)
Mammographic breast density according to BI-RADS®*	
ACR A (almost entirely fatty)	13 (12.9)
ACR B (scattered areas of fibroglandular density)	51 (50.5)
ACR C (heterogeneously dense)	30 (29.7)
ACR D (extremely dense)	7 (6.9)
Previous surgery of the contralateral breast*	15 (14.9)
Positive family history of breast cancer*	16 (15.8)

*Values are absolute frequencies. Relative frequencies are given as percentages in parentheses. Percentages are rounded. **Values are means with standard deviation in parentheses

The *p* values are based on the *χ*^2^ test (*) or *t* test (**)

In total, 101 patients received both tomosynthesis and ABUS scans, 58 in mediolateral-oblique (MLO), 42 in mediolateral (ML), and one in craniocaudal (CC) projection, and were included in the analysis. The applied compression force was on average 106.30 ± 22.47 N and resulted in an averaged compression thickness of 51.83 ± 12.71 mm. The scanning time of ABUS ranged between 40 and 60 s, depending on the breast volume. The total time of performing ABUS and tomosynthesis ranged from 90 to 130 s. The average time for the interpretation of both ABUS and tomosynthesis images was 277 ± 113 s.

### Coverage

The median breast area measured in tomosynthesis was 162.54 cm^2^ (125.25; 198.90) and 123.37 cm^2^ (92.36; 169.42) in ABUS with a median coverage in ABUS of 80.0% (75.0; 86.0) of the tomosynthesis coverage. The area covered in tomosynthesis was significantly larger than that in ABUS with a median difference of 31.57 cm^2^ (23.63; 42.89) (*p* < 0.001, 95% CI [17.74; 21.19]).

### Image quality

All cases were rated as at least category 2, so tissue structures were identifiable in all cases with a median quality of 3 (lower quality compared to HHUS, but well-distinguishable tissue structures). In 86 of 101 cases (85.1%), ABUS image quality was rated as diagnostically useful. In 18 of 101 scans (17.8%), image quality was described as being close to HHUS (category 4). None of the scans was rated as 5 (equal or higher quality than HHUS). There was no significant difference in the rating of image quality over the three projections (*p* = 0.29, see Table 2).

**Table 2:** Image quality per projection

		Projection			*p* value*
		CC	MLO	ML	
Quality	1	0 (0.0)	0 (0.0)	0 (0.0)	0.29
	2	0 (0.0)	6 (10.3)	9 (21.4)	0.29
	3	1 (100.0)	40 (69.0)	27 (64.3)	0.29
	4	0 (0.0)	12 (20.7)	6 (14.3)	0.29
	5	0 (0.0)	0 (0.0)	0 (0.0)	0.29
Total		1	58	42	

The values are absolute frequencies. Numbers in parentheses are column percentages. Percentages are rounded. *The *p* value is based on the Fisher’s exact test under exclusion of the column “CC projection”. *CC* craniocaudal, *MLO* mediolateral-oblique, *ML* mediolateral

### Detection of breast lesions using the FUSION-X-US-II prototype

After the standard diagnostic workup in the breast unit, 53 of 101 cases (52.5%) were classified as likely benign (BI-RADS® 1–3). Forty-eight of 101 cases (47.5%) were classified as unclear or suspicious (BI-RADS® 4–5) with the recommendation for histological confirmation by core-cut biopsy, which was performed in 47 of 101 patients (46.5%). Thirty-four carcinomas (34/101, 33.7%) were diagnosed in the study population (for tumor characteristics, see Table 3).

**Table 3:** Tumor characteristics

Number of carcinoma	34
Palpability	26 (76.5%)
Histopathological type	
Non special type	26 (76.5%)
Invasive lobular carcinoma	7 (20.6%)
Papillary carcinoma	1 (2.9%)
Tumor biology	
Luminal	27 (79.4%)
HER2 neu positive	4 (11.8%)
Triple negative	3 (8.8%)
Grading	
1	3 (8.8%)
2	25 (73.5%)
3	6 (17.6%)

The values given are absolute frequencies. The values in parentheses are relative frequencies

**Fig. 1:**
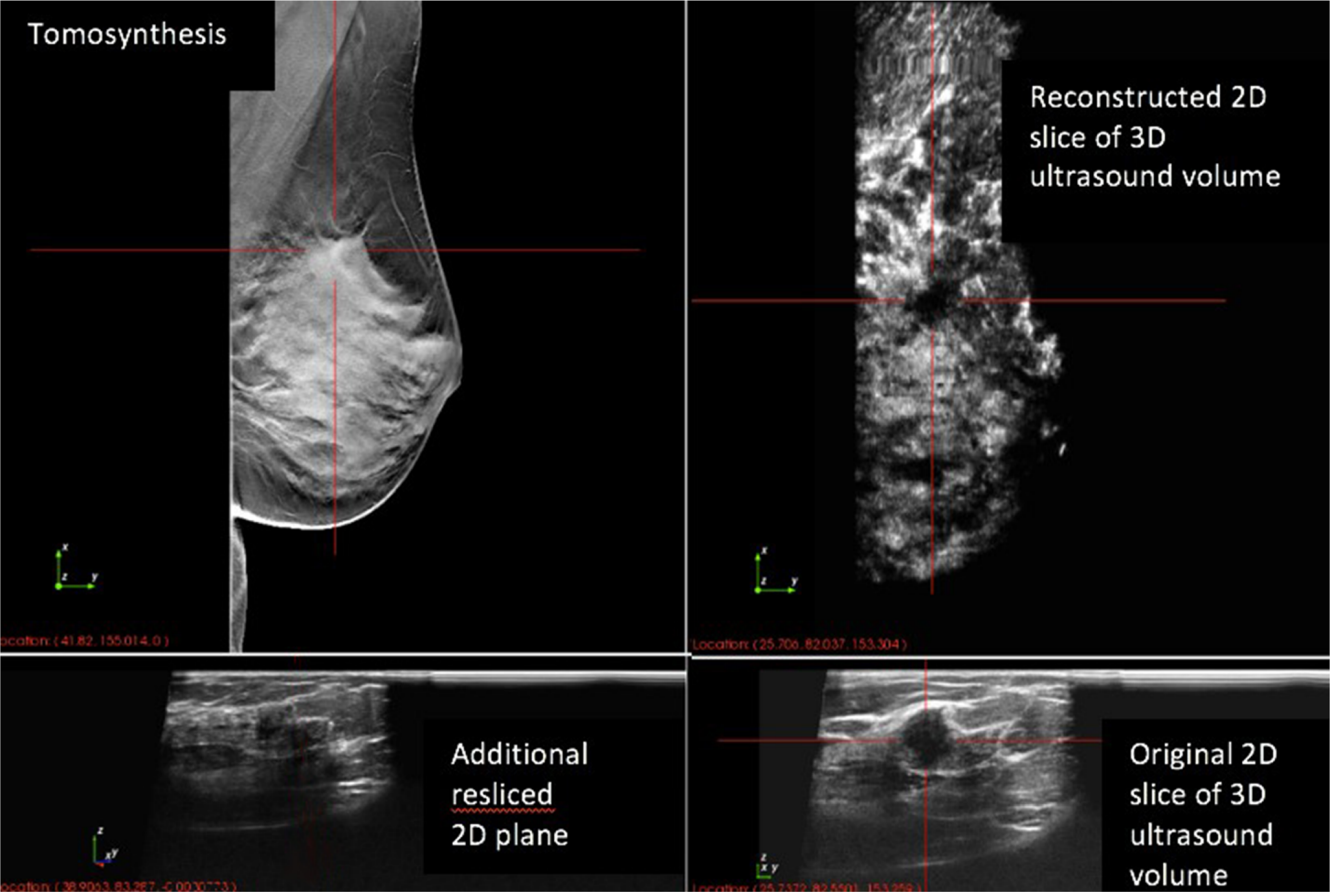
Mammographically and sonographically suspicious lesion in the correlating images of tomosynthesis and ABUS. The patient presented with a palpable lesion. In the standard diagnostic workup, the lesions were highly suspicious in tomosynthesis and hand-held ultrasound. Histology confirmed the diagnosis of invasive carcinoma (NST, ER+, PR−, Her2 neu−, G3, Ki-67 90%).

In the study setting using only the prototype ABUS and tomosynthesis images, 33 of the 34 carcinomas (97.1%) were identified and classified as suspicious (BI-RADS® 4 or 5) or unclear with the need for further diagnostic workup (BI-RADS® 0). Twenty-six of these 33 carcinomas (78.8%) were described in tomosynthesis and ABUS (see Fig. 1), six (18.2%) only in tomosynthesis, and one carcinoma (3.0%) only in ABUS. Descriptively, there seems to be no significant correlation between the mammographic breast density and missed cancers at tomosynthesis or ABUS (see Table 4), but the subgroups were too small for statistically meaningful results.

**Table 4:** Cancer detection through ABUS and tomosynthesis by subgroups of mammographic breast density according to ACR

	Breast density according to ACR			
	A	B	C	D
Number of cases	13	51	30	7
Number of carcinomas	6	19	9	0
Incidence rate	460/1000	370/1000	300/1000	0/1000
Detected with ABUS	5 (83%)	13 (68%)	9 (100%)	0 (0%)
Detected with tomosynthesis	5 (83%)	19 (100%)	8 (89%)	0 (0%)

The values given are absolute frequencies. The values in parentheses are relative frequencies

Twenty-two of 27 carcinomas (81.5%) detected in ABUS were completely visualized, two of 27 carcinomas (7.4%) were partly visualized due to proximity to the thoracic wall. Three of 27 carcinomas (11.1%) were classified as BI-RADS® 0 due to low image quality (category 2). One lesion was described in ABUS as suspicious (BI-RADS® 4) and was not seen in tomosynthesis. A core-cut biopsy confirmed the diagnosis of an invasive lobular carcinoma in the lesion not seen in tomosynthesis. In three cases, which were histologically confirmed as non-special type carcinoma (NST), ABUS led to a correct upgrading of the BI-RADS® classification of tomosynthesis: twice from BI-RADS® 4b to BI-RADS® 5 and once from BI-RADS® 4a to BI-RADS® 4b.

Retrospective evaluation (by the unblinded physician in knowledge of the standard diagnostic workup) of the seven carcinomas not detected in ABUS revealed that five carcinomas (71.4%) were located outside of the area covered by the ABUS images. One of these carcinomas was not detected, by neither ABUS nor tomosynthesis. Two of seven carcinomas (28.6%) were not described in the interpretation of ABUS images in the study setting but could be localized in a retrospective second-look evaluation as discreet architectural distortions.

Sixty-seven of 101 cases (66.3%) were classified as unsuspicious in the standard diagnostic workup, containing no or surely benign lesions (BI-RADS® 1–2, *n* = 41), morphologically likely benign lesions with recommendation for follow-up (BI-RADS® 3, *n* = 12), or were histologically benign after core-cut biopsy had been recommended based on imaging (BI-RADS® 4, *n* = 14). One patient declined the biopsy. Ten of the twelve patients with recommendation for follow-up showed no evidence for malignant disease in the 12 months follow-up at our breast unit. Two patients failed to follow-up.

Forty-two of the unsuspicious lesions (BI-RADS® 1–3) were rated concordantly in the standard diagnostic workup and the study setting. In six cases, lesions were classified as unclear in tomosynthesis (BI-RADS® 0) in the study setting, but could be correctly downgraded through ABUS (BI-RADS® 2). Further, 19 lesions were rated as BI-RADS® 3 or 4 in both the standard diagnostic workup and the study setting. In this group, four cases were classified as BI-RADS® 4 in tomosynthesis and as BI-RADS® 3 in ABUS in the study setting. Formally, ABUS led to a correct downgrading of these cases in the study setting.

Twelve cases were described as unclear or suspicious (BI-RADS® 0 or 4) in the study setting, but were unsuspicious (BI-RADS® 1–3) in the standard diagnostic workup.

Overall, the combined performance of tomosynthesis and ABUS led to a sensitivity of 97.1% (95% CI [91.4; 100]) and a specificity of 59.7% (95% CI [48.0; 71.4]) in the study setting (see Table 5).

**Table 5:** Classification of detected lesions with the FUSION-X-US-II prototype compared with the standard diagnostic workup (gold standard)

		FUSION-X-US-II prototype		
		Unclear/suspicious	Unsuspicious	Total
Standard diagnostic workup (gold standard)	Malignant	33	1	34
	Benign	27	40	67
	Total	60	41	101

The values are absolute frequencies

## Discussion

This is the first larger prospective cohort study on the use of a prototype combining ABUS and tomosynthesis in a clinical setting. The prototype worked technically reliable in most cases and the completed scans showed a high detection rate of carcinomas (33 of the 34 carcinomas, 97.1%). The one carcinoma missed by the FUSION-X-US-II prototype was localized close to the thoracic wall in HHUS in the standard diagnostic workup. ABUS coverage of the breast in the FUSION-X-US-II prototype was improved by 21% compared to the previous prototype. Besides this technical aspect, the present study was performed in a much larger cohort (*n* = 101 vs. *n* = 30) than the FUSION-X-US-I study [26]. Thus, the results in the present study add new evidence on the applicability of the hybrid prototype.

### Technical function, clinical workflow, and patient comfort

The prototype completed both ABUS and tomosynthesis scans in 66.4% of all cases, while tomosynthesis was completed in all cases. Since some minor technical defects could already be fixed while the device was set up in the breast unit, it can be assumed that a thorough technical revision of the prototype will lead to a further decreased error rate. In clinical terms, the technical difficulties have to be systematically eliminated and do not speak against the clinical use of a hybrid device in general. This shows the potential for a reliable and feasible clinical application if hardware and software errors can be reduced.

The study examination was smoothly implemented in the clinical workflow and was well tolerated by the participants. The breast compression procedure for the radiologic technologist was equivalent to standard tomosynthesis except for the easily applicable, additional air cushion. The performance of both tomosynthesis and ABUS combined was a fast procedure with a total scanning time of 90 to 130 s. Regarding the interpretation time of the images (average 277 ± 113 s), one should keep in mind that the cohort had a high prevalence of breast lesions to be described and classified. In a screening-like situation with a lower prevalence of breast lesions, the average time for the interpretation will be substantially shorter.

### Image quality and coverage

Overall, in 85.1% of the cases, ABUS quality was rated category 3 or higher, which means that tissue structures were clearly visible and ABUS images were of diagnostic use. Image quality differed largely depending on the individual breast shape. In general, small breasts were harder to position under the compression paddle and therefore reaching adequate contact with the ultrasound transducer was difficult in some cases. On the hardware level, image resolution of ABUS is limited by the technical specifications of the ultrasound transducer. So far, no specific ultrasound transducer has been designed for the prototype. Adapting current high-end transducers to fit the requirements of the special prototype setup, e.g., regarding penetration depth and resolution, is necessary to reach higher image quality. Importantly, the prototype is not intended to replace high-resolution HHUS as a tool for clarification of unclear or suspicious lesions. It is rather meant to provide additional ABUS in the same orientation as tomosynthesis in a large number of patients who would otherwise not undergo any sonographic examination in a screening setting.

Tomosynthesis and ABUS coverage were sufficient to cover the whole breast of all patients except for one patient with macromastia. In this case, we obtained two scans of both tomosynthesis and ABUS to cover the whole breast. This was no study-specific problem, so we did not exclude the scans in the analysis.

ABUS coverage, which was 66.0% in the FUSION-X-US-I prototype, has been improved and now reaches a median coverage of 80.0% (75.0; 86.0) of the volume covered in tomosynthesis [26]. The increase of coverage especially at the mammillary area can be attributed to the positive effect of the air cushion. A large part of the missing breast area is due to the blind gap of approximately 1 cm towards the pectoralis muscle caused by the housing of the ABUS device. To reduce this shortcoming, a dedicated ultrasound transducer with optimized housing and position of active ultrasound elements will be tested in future prototypes.

### Detection of breast lesions using the FUSION-X-US-II prototype

**Fig. 2:**
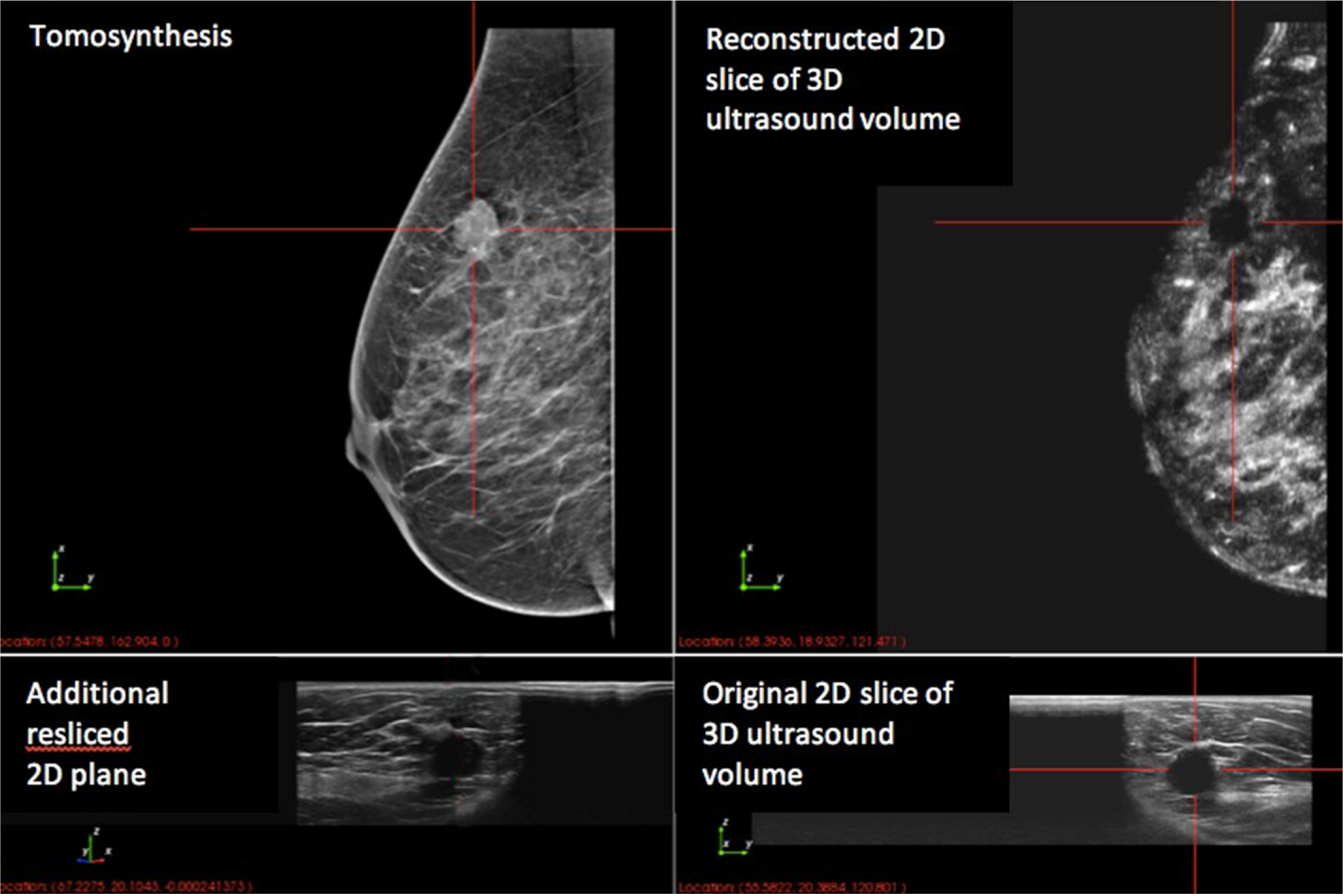
Additional benefit of ABUS: with tomosynthesis, the lesion cannot be seen clearly. In ABUS, the lesion is sonographically suspicious. Histology confirmed the diagnosis of invasive carcinoma (NST, ER+, PR+, Her2 neu−, G1, Ki-67 15%).

The prototype showed a high detection rate of carcinomas (33 of the 34 carcinomas, 97.1%). One of the carcinomas was only seen in ABUS and not in tomosynthesis. In this case, the combined analysis of ABUS and tomosynthesis showed a clear advantage over tomosynthesis alone (see Fig. 2). In the standard diagnostic workup, this carcinoma presented as a palpable lesion with a sonographic correlate in HHUS but no mammographic correlate. Only one carcinoma was missed in the prototype setting. Due to its localization close to the thoracic wall, this lesion was only visible in HHUS in the standard diagnostic workup.

Benign lesions were not assessed systematically in the study, because unsuspicious masses (e.g., cysts or duct ectasia) are not routinely described in detail in the reports of the standard diagnostic workup. Regarding the BI-RADS® classifications on the case level, ABUS in the prototype setting led to a correct downgrading of ten cases, which were falsely described as suspicious or as unclear in tomosynthesis. On the other hand, the prototype decreased the diagnostic precision or formally led to a false upgrading of twelve cases compared to the standard diagnostic workup.

### Clinical implications

This is the first larger prospective cohort study on the use of a prototype combining ABUS and tomosynthesis in a clinical setting. The prevalence of malignant lesions in the study cohort (34 of 101, 33.7%) was high enough to evaluate the detection of malignant lesions using the prototype.

The results of this study show that a combined performance of ABUS and tomosynthesis in a clinical setting using the FUSION-X-US-II prototype is feasible and time efficient. The examination was fully integrated into the clinical workflow, so the clinical applicability of the results is given. Since the examination is performed by a radiologic technologist and the physician can afterwards interpret the images separately, there is a potential cost-saving factor. In the future, automatization and standardization to acquire clinical image data might gain further importance when image recognition algorithms are used to support clinical decision-making. Our analysis on automized and standardized ABUS combined with tomosynthesis is an important step to enable development of such algorithms in the near future.

In most current screening programs, HHUS is only performed in patients with unclear mammographic findings. Hybrid devices like this prototype could potentially allow a higher number of patients to receive supplemental ABUS to mammography/tomosynthesis. Previous studies suggest that a widespread use of ABUS can lead to the detection of additional carcinomas [29], but further evidence for a clinical benefit is needed. ABUS is a potential screening tool and not a substitute for HHUS in a diagnostic situation. Therefore, any unclear or suspicious lesions need to be further examined with HHUS.

## Limitations

The inclusion criteria led to a high prevalence of breast lesions (selection bias), which could be expected by the examiner (observer bias). To limit the observer bias, the examiner was blinded to the results of the standard diagnostic workup. In a screening population with much lower prevalence of breast lesions, the detection rate using the prototype will likely be lower than that in the study setting, which has to be considered regarding the potential applicability of the prototype for screening-like situations.

We did not separate the readings of tomosynthesis and ABUS, because we tried to simulate a clinical workflow as realistically as possible where one would always aim to have as much comprehensive information available at the same time to give a diagnosis based on all diagnostic procedures. So, only the reading of tomosynthesis alone can clearly be separated from ABUS, while the reading of ABUS might have had a recall bias.

As the scans were evaluated by only one observer, we were not able to calculate interobserver agreement, which is an important question regarding the applicability in screening-like situations and therefore should be assessed in the following studies. The image quality was also only assessed subjectively by one examiner, whose impression of image quality is influenced by several factors, e.g., image resolution, presence of artefacts, coverage, and personal experience leading.

Similarly, the assessment of breast coverage is difficult, because there was no tool to measure the three-dimensional extension of each breast. Therefore, the measurements of tomosynthesis were used as a reference standard for the coverage of ABUS.

## Conclusions

Overall, this study has shown that a combined performance of tomosynthesis and ABUS with the FUSION-X-US-II prototype can be successfully implemented in a clinical workflow. Malignant lesions were accurately detected. For a clinical application, image quality and coverage of the prototype need to be improved. Further studies are needed to evaluate the potential benefit in a screening collective.

## Abbreviations

ABUS: 3D automated breast ultrasound
CC: Craniocaudal
ER: Estrogen receptor
HHUS: Hand-held ultrasound
HIPPA: Health Information Portability and Accountability Act of 1996
ML: Mediolateral
MLO: Mediolateral-oblique
PR: Progesterone receptor
